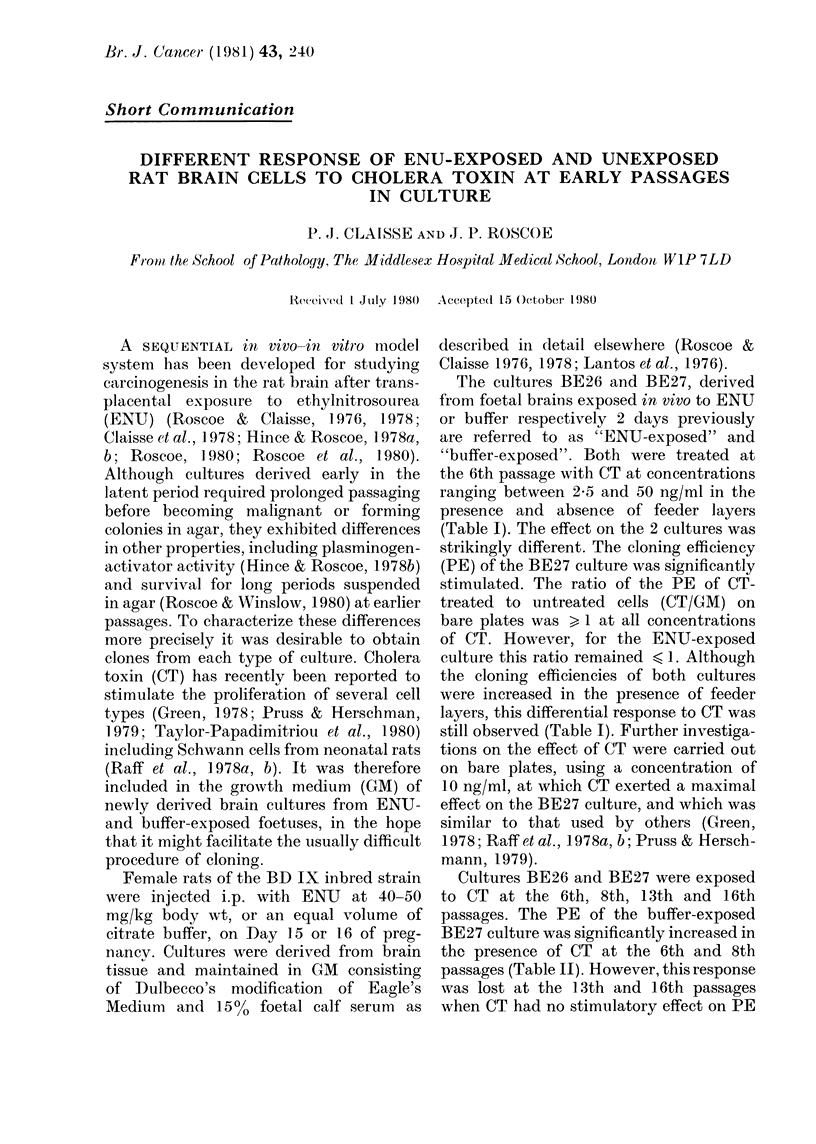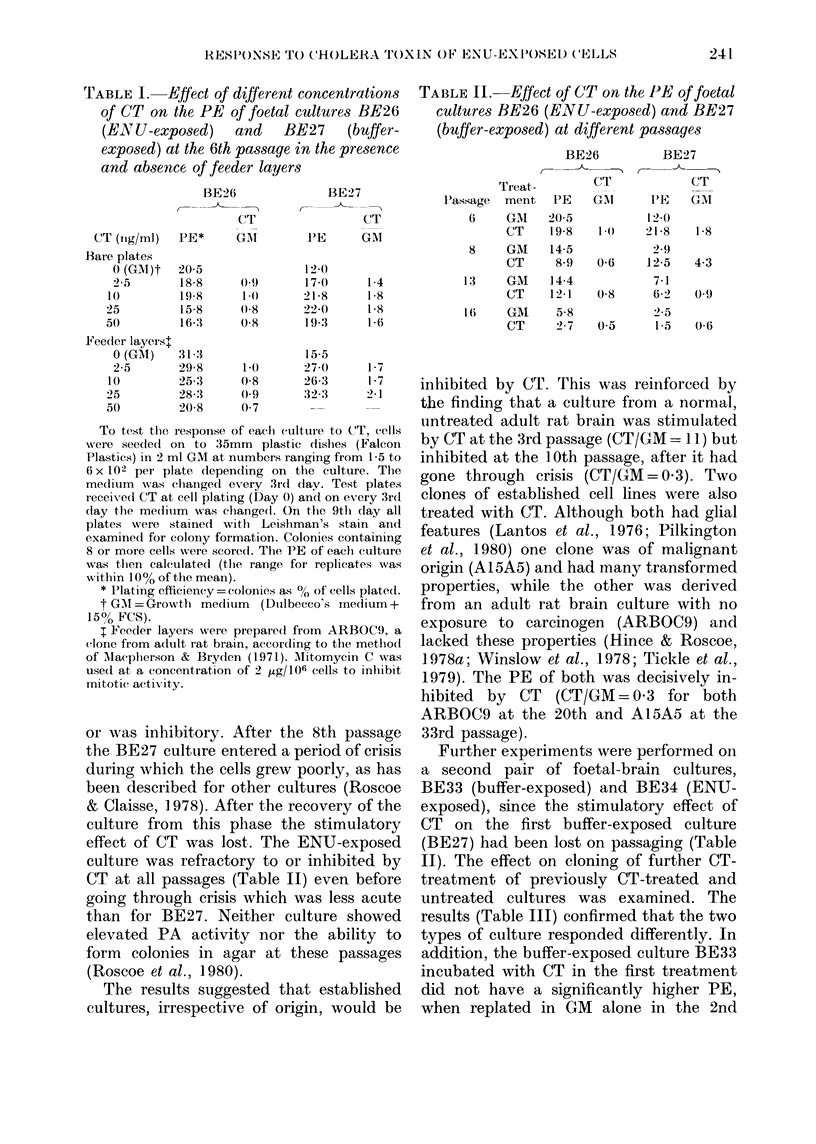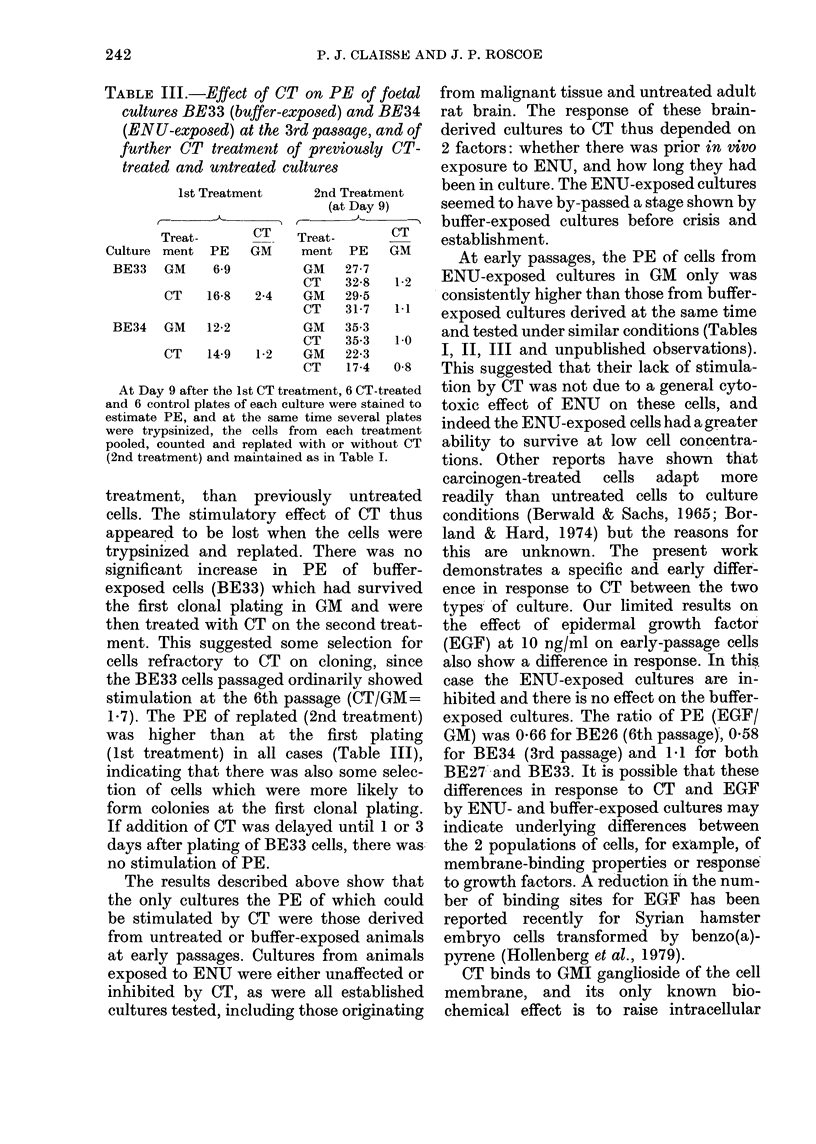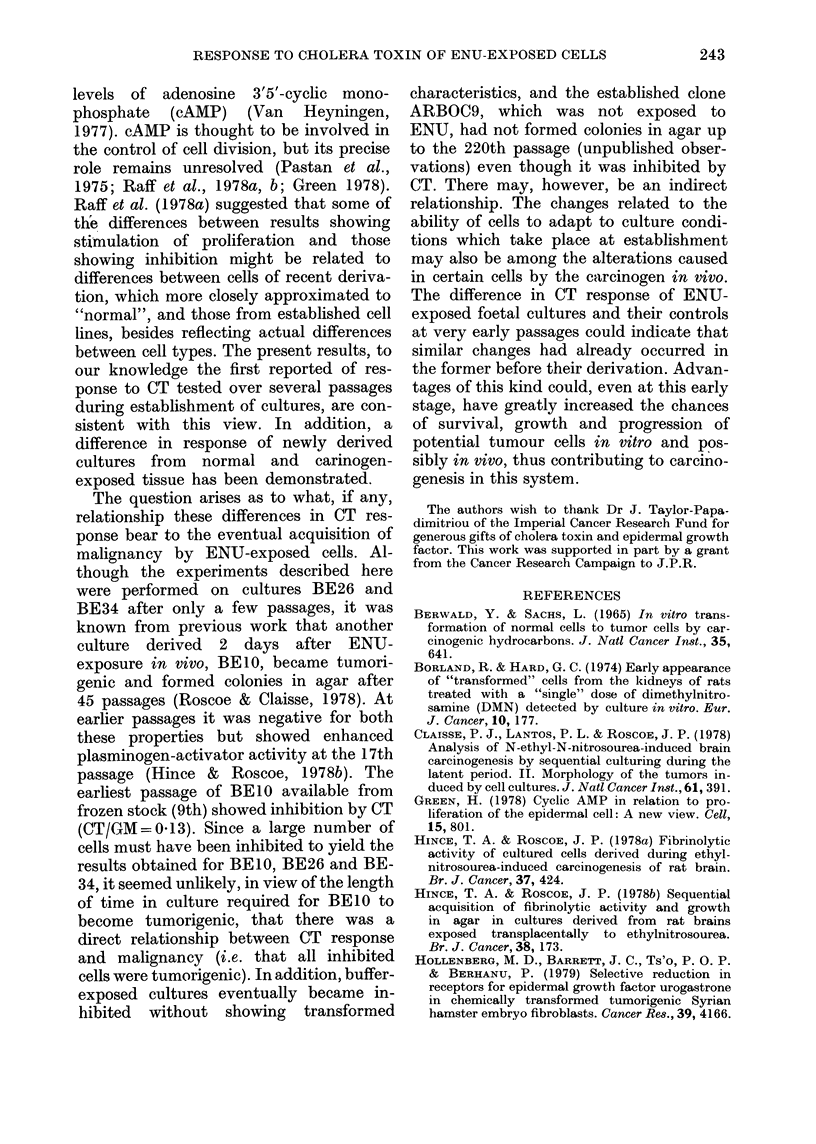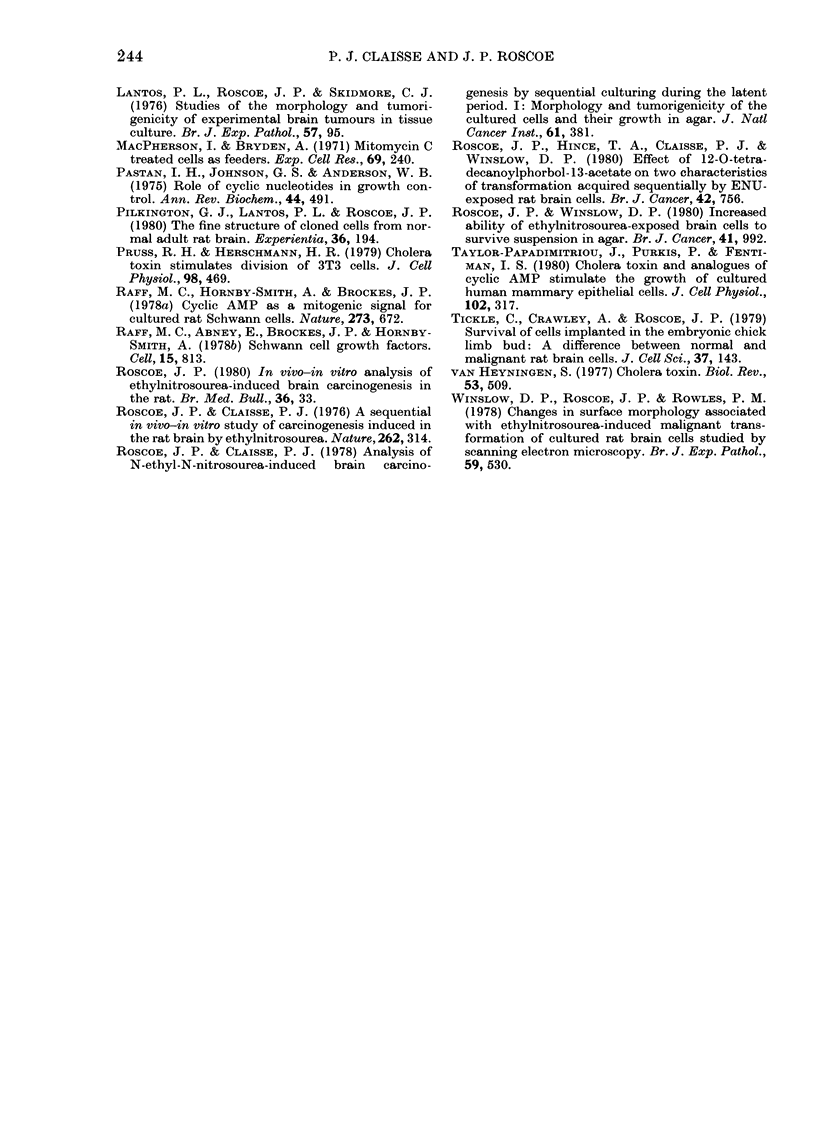# Different response of ENU-exposed and unexposed rat brain cells to cholera toxin at early passages in culture.

**DOI:** 10.1038/bjc.1981.36

**Published:** 1981-02

**Authors:** P. J. Claisse, J. P. Roscoe


Br. J. Cancer (1981) 43, 240

*Short Communication*

### DIFFERENT RESPONSE OF ENU-EXPOSED AND UNEXPOSED RAT BRAIN CELLS TO CHOLERA TOXIN AT EARLY PASSAGES IN CULTURE

P. J. CLAISSE AND J. P. ROSCOE

*From the School of Pathology, The Middlesex Hospital Medical School, London W1P 7LD*

Received 1 July 1980 Accepted 15 October 1980

A SEQUENTIAL *in vivo–in vitro* model system has been developed for studying carcinogenesis in the rat brain after transplacental exposure to ethylnitrosourea (ENU) (Roscoe & Claisse, 1976, 1978; Claisse *et al.*, 1978; Hince & Roscoe, 1978*a*, *b*; Roscoe, 1980; Roscoe *et al.*, 1980). Although cultures derived early in the latent period required prolonged passaging before becoming malignant or forming colonies in agar, they exhibited differences in other properties, including plasminogen-activator activity (Hince & Roscoe, 1978*b*) and survival for long periods suspended in agar (Roscoe & Winslow, 1980) at earlier passages. To characterize these differences more precisely it was desirable to obtain clones from each type of culture. Cholera toxin (CT) has recently been reported to stimulate the proliferation of several cell types (Green, 1978; Pruss & Herschman, 1979; Taylor-Papadimitriou *et al.*, 1980) including Schwann cells from neonatal rats (Raff *et al.*, 1978*a*, *b*). It was therefore included in the growth medium (GM) of newly derived brain cultures from ENU- and buffer-exposed foetuses, in the hope that it might facilitate the usually difficult procedure of cloning.

Female rats of the BD IX inbred strain were injected i.p. with ENU at 40–50 mg/kg body wt, or an equal volume of citrate buffer, on Day 15 or 16 of pregnancy. Cultures were derived from brain tissue and maintained in GM consisting of Dulbecco's modification of Eagle's Medium and 15% foetal calf serum as described in detail elsewhere (Roscoe & Claisse 1976, 1978; Lantos *et al.*, 1976).

The cultures BE26 and BE27, derived from foetal brains exposed *in vivo* to ENU or buffer respectively 2 days previously are referred to as "ENU-exposed" and "buffer-exposed". Both were treated at the 6th passage with CT at concentrations ranging between 2·5 and 50 ng/ml in the presence and absence of feeder layers (Table I). The effect on the 2 cultures was strikingly different. The cloning efficiency (PE) of the BE27 culture was significantly stimulated. The ratio of the PE of CT-treated to untreated cells (CT/GM) on bare plates was $\geqslant 1$ at all concentrations of CT. However, for the ENU-exposed culture this ratio remained $\leqslant 1$. Although the cloning efficiencies of both cultures were increased in the presence of feeder layers, this differential response to CT was still observed (Table I). Further investigations on the effect of CT were carried out on bare plates, using a concentration of 10 ng/ml, at which CT exerted a maximal effect on the BE27 culture, and which was similar to that used by others (Green, 1978; Raff *et al.*, 1978*a, b*; Pruss & Herschmann, 1979).

Cultures BE26 and BE27 were exposed to CT at the 6th, 8th, 13th and 16th passages. The PE of the buffer-exposed BE27 culture was significantly increased in the presence of CT at the 6th and 8th passages (Table II). However, this response was lost at the 13th and 16th passages when CT had no stimulatory effect on PE

TABLE I.—*Effect of different concentrations of CT on the PE of foetal cultures BE26 (ENU-exposed) and BE27 (buffer-exposed) at the 6th passage in the presence and absence of feeder layers*

| | BE26 | | BE27 | |
|---|---|---|---|---|
| CT (ng/ml) | PE* | CT/GM | PE | CT/GM |
| Bare plates | | | | |
| 0 (GM)† | 20·5 | | 12·0 | |
| 2·5 | 18·8 | 0·9 | 17·0 | 1·4 |
| 10 | 19·8 | 1·0 | 21·8 | 1·8 |
| 25 | 15·8 | 0·8 | 22·0 | 1·8 |
| 50 | 16·3 | 0·8 | 19·3 | 1·6 |
| Feeder layers‡ | | | | |
| 0 (GM) | 31·3 | | 15·5 | |
| 2·5 | 29·8 | 1·0 | 27·0 | 1·7 |
| 10 | 25·3 | 0·8 | 26·3 | 1·7 |
| 25 | 28·3 | 0·9 | 32·3 | 2·1 |
| 50 | 20·8 | 0·7 | — | — |

To test the response of each culture to CT, cells were seeded on to 35mm plastic dishes (Falcon Plastics) in 2 ml GM at numbers ranging from 1·5 to $6 \times 10^2$ per plate depending on the culture. The medium was changed every 3rd day. Test plates received CT at cell plating (Day 0) and on every 3rd day the medium was changed. On the 9th day all plates were stained with Leishman's stain and examined for colony formation. Colonies containing 8 or more cells were scored. The PE of each culture was then calculated (the range for replicates was within 10% of the mean).

* Plating efficiency = colonies as % of cells plated.

† GM = Growth medium (Dulbecco's medium + 15% FCS).

‡ Feeder layers were prepared from ARBOC9, a clone from adult rat brain, according to the method of Macpherson & Bryden (1971). Mitomycin C was used at a concentration of 2 $\mu g/10^6$ cells to inhibit mitotic activity.

or was inhibitory. After the 8th passage the BE27 culture entered a period of crisis during which the cells grew poorly, as has been described for other cultures (Roscoe & Claisse, 1978). After the recovery of the culture from this phase the stimulatory effect of CT was lost. The ENU-exposed culture was refractory to or inhibited by CT at all passages (Table II) even before going through crisis which was less acute than for BE27. Neither culture showed elevated PA activity nor the ability to form colonies in agar at these passages (Roscoe *et al.*, 1980).

The results suggested that established cultures, irrespective of origin, would be

TABLE II.—*Effect of CT on the PE of foetal cultures BE26 (ENU-exposed) and BE27 (buffer-exposed) at different passages*

| | | BE26 | | BE27 | |
|---|---|---|---|---|---|
| Passage | Treat-ment | PE | CT/GM | PE | CT/GM |
| 6 | GM | 20·5 | | 12·0 | |
| | CT | 19·8 | 1·0 | 21·8 | 1·8 |
| 8 | GM | 14·5 | | 2·9 | |
| | CT | 8·9 | 0·6 | 12·5 | 4·3 |
| 13 | GM | 14·4 | | 7·1 | |
| | CT | 12·1 | 0·8 | 6·2 | 0·9 |
| 16 | GM | 5·8 | | 2·5 | |
| | CT | 2·7 | 0·5 | 1·5 | 0·6 |

inhibited by CT. This was reinforced by the finding that a culture from a normal, untreated adult rat brain was stimulated by CT at the 3rd passage (CT/GM = 11) but inhibited at the 10th passage, after it had gone through crisis (CT/GM = 0·3). Two clones of established cell lines were also treated with CT. Although both had glial features (Lantos *et al.*, 1976; Pilkington *et al.*, 1980) one clone was of malignant origin (A15A5) and had many transformed properties, while the other was derived from an adult rat brain culture with no exposure to carcinogen (ARBOC9) and lacked these properties (Hince & Roscoe, 1978*a*; Winslow *et al.*, 1978; Tickle *et al.*, 1979). The PE of both was decisively inhibited by CT (CT/GM = 0·3 for both ARBOC9 at the 20th and A15A5 at the 33rd passage).

Further experiments were performed on a second pair of foetal-brain cultures, BE33 (buffer-exposed) and BE34 (ENU-exposed), since the stimulatory effect of CT on the first buffer-exposed culture (BE27) had been lost on passaging (Table II). The effect on cloning of further CT-treatment of previously CT-treated and untreated cultures was examined. The results (Table III) confirmed that the two types of culture responded differently. In addition, the buffer-exposed culture BE33 incubated with CT in the first treatment did not have a significantly higher PE, when replated in GM alone in the 2nd

TABLE III.—*Effect of CT on PE of foetal cultures BE33 (buffer-exposed) and BE34 (ENU-exposed) at the 3rd passage, and of further CT treatment of previously CT-treated and untreated cultures*

| | 1st Treatment | | | 2nd Treatment (at Day 9) | | |
|---|---|---|---|---|---|---|
| Culture | Treatment | PE | CT/GM | Treatment | PE | CT/GM |
| BE33 | GM | 6·9 | | GM | 27·7 | |
| | | | | CT | 32·8 | 1·2 |
| | CT | 16·8 | 2·4 | GM | 29·5 | |
| | | | | CT | 31·7 | 1·1 |
| BE34 | GM | 12·2 | | GM | 35·3 | |
| | | | | CT | 35·3 | 1·0 |
| | CT | 14·9 | 1·2 | GM | 22·3 | |
| | | | | CT | 17·4 | 0·8 |

At Day 9 after the 1st CT treatment, 6 CT-treated and 6 control plates of each culture were stained to estimate PE, and at the same time several plates were trypsinized, the cells from each treatment pooled, counted and replated with or without CT (2nd treatment) and maintained as in Table I.

treatment, than previously untreated cells. The stimulatory effect of CT thus appeared to be lost when the cells were trypsinized and replated. There was no significant increase in PE of buffer-exposed cells (BE33) which had survived the first clonal plating in GM and were then treated with CT on the second treatment. This suggested some selection for cells refractory to CT on cloning, since the BE33 cells passaged ordinarily showed stimulation at the 6th passage (CT/GM= 1·7). The PE of replated (2nd treatment) was higher than at the first plating (1st treatment) in all cases (Table III), indicating that there was also some selection of cells which were more likely to form colonies at the first clonal plating. If addition of CT was delayed until 1 or 3 days after plating of BE33 cells, there was no stimulation of PE.

The results described above show that the only cultures the PE of which could be stimulated by CT were those derived from untreated or buffer-exposed animals at early passages. Cultures from animals exposed to ENU were either unaffected or inhibited by CT, as were all established cultures tested, including those originating from malignant tissue and untreated adult rat brain. The response of these brain-derived cultures to CT thus depended on 2 factors: whether there was prior *in vivo* exposure to ENU, and how long they had been in culture. The ENU-exposed cultures seemed to have by-passed a stage shown by buffer-exposed cultures before crisis and establishment.

At early passages, the PE of cells from ENU-exposed cultures in GM only was consistently higher than those from buffer-exposed cultures derived at the same time and tested under similar conditions (Tables I, II, III and unpublished observations). This suggested that their lack of stimulation by CT was not due to a general cytotoxic effect of ENU on these cells, and indeed the ENU-exposed cells had a greater ability to survive at low cell concentrations. Other reports have shown that carcinogen-treated cells adapt more readily than untreated cells to culture conditions (Berwald & Sachs, 1965; Borland & Hard, 1974) but the reasons for this are unknown. The present work demonstrates a specific and early difference in response to CT between the two types of culture. Our limited results on the effect of epidermal growth factor (EGF) at 10 ng/ml on early-passage cells also show a difference in response. In this case the ENU-exposed cultures are inhibited and there is no effect on the buffer-exposed cultures. The ratio of PE (EGF/GM) was 0·66 for BE26 (6th passage), 0·58 for BE34 (3rd passage) and 1·1 for both BE27 and BE33. It is possible that these differences in response to CT and EGF by ENU- and buffer-exposed cultures may indicate underlying differences between the 2 populations of cells, for example, of membrane-binding properties or response to growth factors. A reduction in the number of binding sites for EGF has been reported recently for Syrian hamster embryo cells transformed by benzo(a)-pyrene (Hollenberg *et al.*, 1979).

CT binds to GMI ganglioside of the cell membrane, and its only known biochemical effect is to raise intracellular levels of adenosine 3′5′-cyclic monophosphate (cAMP) (Van Heyningen, 1977). cAMP is thought to be involved in the control of cell division, but its precise role remains unresolved (Pastan *et al.*, 1975; Raff *et al.*, 1978*a, b*; Green 1978). Raff *et al.* (1978*a*) suggested that some of the differences between results showing stimulation of proliferation and those showing inhibition might be related to differences between cells of recent derivation, which more closely approximated to "normal", and those from established cell lines, besides reflecting actual differences between cell types. The present results, to our knowledge the first reported of response to CT tested over several passages during establishment of cultures, are consistent with this view. In addition, a difference in response of newly derived cultures from normal and carinogen-exposed tissue has been demonstrated.

The question arises as to what, if any, relationship these differences in CT response bear to the eventual acquisition of malignancy by ENU-exposed cells. Although the experiments described here were performed on cultures BE26 and BE34 after only a few passages, it was known from previous work that another culture derived 2 days after ENU-exposure *in vivo*, BE10, became tumorigenic and formed colonies in agar after 45 passages (Roscoe & Claisse, 1978). At earlier passages it was negative for both these properties but showed enhanced plasminogen-activator activity at the 17th passage (Hince & Roscoe, 1978*b*). The earliest passage of BE10 available from frozen stock (9th) showed inhibition by CT (CT/GM = 0·13). Since a large number of cells must have been inhibited to yield the results obtained for BE10, BE26 and BE-34, it seemed unlikely, in view of the length of time in culture required for BE10 to become tumorigenic, that there was a direct relationship between CT response and malignancy (*i.e.* that all inhibited cells were tumorigenic). In addition, buffer-exposed cultures eventually became inhibited without showing transformed characteristics, and the established clone ARBOC9, which was not exposed to ENU, had not formed colonies in agar up to the 220th passage (unpublished observations) even though it was inhibited by CT. There may, however, be an indirect relationship. The changes related to the ability of cells to adapt to culture conditions which take place at establishment may also be among the alterations caused in certain cells by the carcinogen *in vivo*. The difference in CT response of ENU-exposed foetal cultures and their controls at very early passages could indicate that similar changes had already occurred in the former before their derivation. Advantages of this kind could, even at this early stage, have greatly increased the chances of survival, growth and progression of potential tumour cells *in vitro* and possibly *in vivo*, thus contributing to carcinogenesis in this system.

The authors wish to thank Dr J. Taylor-Papadimitriou of the Imperial Cancer Research Fund for generous gifts of cholera toxin and epidermal growth factor. This work was supported in part by a grant from the Cancer Research Campaign to J.P.R.

REFERENCES

BERWALD, Y. & SACHS, L. (1965) *In vitro* transformation of normal cells to tumor cells by carcinogenic hydrocarbons. *J. Natl Cancer Inst.*, **35**, 641.

BORLAND, R. & HARD, G. C. (1974) Early appearance of "transformed" cells from the kidneys of rats treated with a "single" dose of dimethylnitrosamine (DMN) detected by culture *in vitro*. *Eur. J. Cancer*, **10**, 177.

CLAISSE, P. J., LANTOS, P. L. & ROSCOE, J. P. (1978) Analysis of N-ethyl-N-nitrosourea-induced brain carcinogenesis by sequential culturing during the latent period. II. Morphology of the tumors induced by cell cultures. *J. Natl Cancer Inst.*, **61**, 391.

GREEN, H. (1978) Cyclic AMP in relation to proliferation of the epidermal cell: A new view. *Cell*, **15**, 801.

HINCE, T. A. & ROSCOE, J. P. (1978*a*) Fibrinolytic activity of cultured cells derived during ethylnitrosourea-induced carcinogenesis of rat brain. *Br. J. Cancer*, **37**, 424.

HINCE, T. A. & ROSCOE, J. P. (1978*b*) Sequential acquisition of fibrinolytic activity and growth in agar in cultures derived from rat brains exposed transplacentally to ethylnitrosourea. *Br. J. Cancer*, **38**, 173.

HOLLENBERG, M. D., BARRETT, J. C., TS'O, P. O. P. & BERHANU, P. (1979) Selective reduction in receptors for epidermal growth factor urogastrone in chemically transformed tumorigenic Syrian hamster embryo fibroblasts. *Cancer Res.*, **39**, 4166.

LANTOS, P. L., ROSCOE, J. P. & SKIDMORE, C. J. (1976) Studies of the morphology and tumorigenicity of experimental brain tumours in tissue culture. *Br. J. Exp. Pathol.*, **57**, 95.

MACPHERSON, I. & BRYDEN, A. (1971) Mitomycin C treated cells as feeders. *Exp. Cell Res.*, **69**, 240.

PASTAN, I. H., JOHNSON, G. S. & ANDERSON, W. B. (1975) Role of cyclic nucleotides in growth control. *Ann. Rev. Biochem.*, **44**, 491.

PILKINGTON, G. J., LANTOS, P. L. & ROSCOE, J. P. (1980) The fine structure of cloned cells from normal adult rat brain. *Experientia*, **36**, 194.

PRUSS, R. H. & HERSCHMANN, H. R. (1979) Cholera toxin stimulates division of 3T3 cells. *J. Cell Physiol.*, **98**, 469.

RAFF, M. C., HORNBY-SMITH, A. & BROCKES, J. P. (1978*a*) Cyclic AMP as a mitogenic signal for cultured rat Schwann cells. *Nature*, **273**, 672.

RAFF, M. C., ABNEY, E., BROCKES, J. P. & HORNBY-SMITH, A. (1978*b*) Schwann cell growth factors. *Cell*, **15**, 813.

ROSCOE, J. P. (1980) *In vivo–in vitro* analysis of ethylnitrosourea-induced brain carcinogenesis in the rat. *Br. Med. Bull.*, **36**, 33.

ROSCOE, J. P. & CLAISSE, P. J. (1976) A sequential *in vivo–in vitro* study of carcinogenesis induced in the rat brain by ethylnitrosourea. *Nature*, **262**, 314.

ROSCOE, J. P. & CLAISSE, P. J. (1978) Analysis of N-ethyl-N-nitrosourea-induced brain carcinogenesis by sequential culturing during the latent period. I: Morphology and tumorigenicity of the cultured cells and their growth in agar. *J. Natl Cancer Inst.*, **61**, 381.

ROSCOE, J. P., HINCE, T. A., CLAISSE, P. J. & WINSLOW, D. P. (1980) Effect of 12-O-tetradecanoylphorbol-13-acetate on two characteristics of transformation acquired sequentially by ENU-exposed rat brain cells. *Br. J. Cancer*, **42**, 756.

ROSCOE, J. P. & WINSLOW, D. P. (1980) Increased ability of ethylnitrosourea-exposed brain cells to survive suspension in agar. *Br. J. Cancer*, **41**, 992.

TAYLOR-PAPADIMITRIOU, J., PURKIS, P. & FENTIMAN, I. S. (1980) Cholera toxin and analogues of cyclic AMP stimulate the growth of cultured human mammary epithelial cells. *J. Cell Physiol.*, **102**, 317.

TICKLE, C., CRAWLEY, A. & ROSCOE, J. P. (1979) Survival of cells implanted in the embryonic chick limb bud: A difference between normal and malignant rat brain cells. *J. Cell Sci.*, **37**, 143.

VAN HEYNINGEN, S. (1977) Cholera toxin. *Biol. Rev.*, **53**, 509.

WINSLOW, D. P., ROSCOE, J. P. & ROWLES, P. M. (1978) Changes in surface morphology associated with ethylnitrosourea-induced malignant transformation of cultured rat brain cells studied by scanning electron microscopy. *Br. J. Exp. Pathol.*, **59**, 530.